# Involuntary Clozapine Treatment: A Systematic Review

**DOI:** 10.1111/acps.70076

**Published:** 2026-02-12

**Authors:** Hélène Verdoux, Alexis Lepetit, Peter F. J. Schulte

**Affiliations:** ^1^ French Clozapine Task Force Bordeaux France; ^2^ Team Pharmacoepidemiology, UMR 1219 Univ. Bordeaux, Inserm, Bordeaux Population Health Research Center Bordeaux France; ^3^ Hospices Civils de Lyon Hôpital des Charpennes Villeurbanne France; ^4^ Dutch Clozapine Collaboration Group Alkmaar the Netherlands; ^5^ Mental Health Services Noord‐Holland‐Noord Alkmaar the Netherlands

**Keywords:** barriers, clozapine, intramuscular, involuntary treatment, nasogastric

## Abstract

**Introduction:**

We aimed to synthesize the information relevant for clinical practice on involuntary clozapine treatment.

**Methods:**

Articles were identified with MEDLINE, Web of Sciences and PsycINFO search from inception through September 2025 (PROSPERO database registration CRD420251234475). We included all articles addressing issues related to involuntary clozapine treatment irrespective of the route of administration, i.e., oral, intra‐muscular (IM) or nasogastric. Data were synthesized narratively.

**Results:**

Of the 29 identified articles, most clinical studies (*n* = 18) on people prescribed involuntary clozapine treatment (*n* = 236) were case reports/series or chart reviews. IM or nasogastric routes were the last‐resort treatment for people with extremely severe psychotic disorders presenting with risky behavior. The decisional process was often lengthy due to the complex legal and ethical issues raised by involuntary treatment and restricted access to the unlicensed IM formulation. In nearly half of cases, the oral route was accepted after the decision to perform IM or nasogastric administration. Pain at the injection site was the most frequent adverse event after IM administration. Transition to this route occurred rapidly in the vast majority of the other cases, most often allowing a dramatic reduction in the severity of target symptoms and coercive measures. Clozapine was maintained orally after the acute phase in the majority of people with involuntary administration.

**Conclusions:**

Although the body of evidence supporting the use of involuntary clozapine treatment is mostly drawn from small observational studies, their findings suggest that this last‐resort option may save the life and promote recovery of people for whom other treatments have failed. Access to IM clozapine is currently restricted in most countries. Whether this barrier to clozapine treatment for severely ill people with impaired decision‐making capacities should be overcome in other countries needs to be further addressed.

**Registration:** PROSPERO database registration CRD420251234475.

## Key Points

- Summations
  ○ Involuntary clozapine treatment may represent the last‐resort option for people with severe psychotic disorders associated with impaired capacity to make health decisions.
  ○ After the prescription of involuntary clozapine treatment, voluntary oral route was rapidly accepted in most cases and subsequently maintained after the acute phase, allowing a striking reduction in target symptom severity and coercive measures.
  ○ The restricted availability of the intramuscular formulation may be considered a barrier to clozapine treatment for some of the most severely ill people with psychosis.
- Limitations
  ○ Information on the benefits and risks of involuntary clozapine prescription and administration is mostly drawn from case reports and retrospective studies of small sample sizes.
  ○ People included in the studies on involuntary clozapine treatment may not be representative since they may present with the most severe conditions or with a favorable outcome.
  ○ Since there is no published data on the pharmacokinetics of intramuscular clozapine, the current recommendations for doses are empirically based on a limited number of cases.

## 1 Introduction

Barriers to clozapine (CLO) initiation are most often related to prescribers' knowledge and attitudes, as well as to the institutional complexity of CLO care organization [1, 2, 3, 4, 5, 6, 7, 8, 9]. The frequency of CLO refusal by people presenting with treatment‐resistant severe mental illnesses (SMI) is often overestimated by health professionals, since most of them accept to initiate CLO after receiving information about its benefit/risk balance [10, 11, 12].

Since antipsychotic resistance is the target indication of CLO, users' information about its benefit/risk balance may be complexified by the lack of remission of psychotic symptoms [13]. Hence, CLO approval may be compromised by an impaired capacity to make an informed decision about optimal treatment because of poor insight, persecutory delusions, or disorganization [14, 15, 16, 17, 18, 19]. In such circumstances, health professionals may face an ethical dilemma: respect the will and preference of people presenting with severe resistant psychotic symptoms and often with a high risk for suicide or assaultive behavior, or decide that CLO should be administered even if coercive measures are required, since this is the optimal therapeutic option [14, 15, 16, 17, 18, 19].

The extensive body of literature on coercive measures in psychiatric care [20, 21] contrast with the small number of studies on the involuntary administration of psychotropic drugs although at least 2%–8% of people admitted to psychiatric care are concerned by such practices [22, 23, 24, 25, 26]. The legislation on involuntary medication administration varies widely between countries [16, 18, 23, 27, 28, 29, 30, 31]. An European multicenter study showed that the most frequent coercive measure in psychiatric inpatients was involuntary medication administration (56%), most frequently involving antipsychotics [23]. A Danish population‐based study found that 16.5% of inpatients with schizophrenia underwent involuntary treatment [27]. Of them, 59% were concerned by involuntary psychotropic drug administration, including antipsychotics in nearly all cases (99.5%).

In contrast to the high frequency of the involuntary administration of antipsychotics [23, 27], involuntary CLO treatment is exceptional [14, 15, 16, 17, 18, 19]. In the Danish study, oral CLO administration concerned 4.8% of all cases of involuntary antipsychotics administration, and only two intra‐muscular (IM) administrations were recorded [27]. The rarity of involuntary CLO treatment is partly explained by the fact that the IM formulation is an unlicensed product currently available only in a few countries [15, 18, 28, 32, 33, 34, 35, 36]. Whether this situation leads to unmet needs in people with SMI remains a matter of debate. Indeed, people with resistant psychotic symptoms have the right to benefit from the optimal treatment when their capacity to make therapeutic decisions is impaired [15, 16, 17, 19, 37]. This potential barrier to CLO treatment deserves further investigation since few prescribers are aware that it may be overcome. Clarifying the indications and the benefit/risk balance of involuntary CLO treatment is also necessary to convince health regulatory agencies to facilitate access to the IM formulation.

## 2 Aims of the Study

The present systematic review sought to synthesize the clinically relevant information on involuntary CLO treatment, irrespective of the route of administration.

## 3 Materials and Methods

### 3.1 Search Strategy

This review was conducted according to the guidelines of the Preferred Reporting Items for Systematic Reviews and Meta‐Analyses (PRISMA) statement [38]. A protocol was published in the PROSPERO database under registration number CRD420251234475. We performed a MEDLINE, Web of Sciences and PsycINFO search from inception through September 2025. We used the term “clozapine” in combination with the search terms (“refusal” OR “consent” OR “coercion” OR “coercive” OR “constraint” OR “forced” OR “enforced” OR “compulsory” OR “involuntary” OR “intramuscular” OR “parenteral” OR “nasogastric”). We also examined related references of selected papers.

Involuntary CLO treatment was defined as the decision to prescribe CLO treatment against the patient's will, regardless of: (i) the route of administration (oral, IM, nasogastric) after the prescription decision; (ii) the use of physical restraint; and (iii) the legal authorization for treatment administration. Thus, this definition included IM or nasogastric CLO administration with or without physical restraint, but also CLO taken orally under the threat of IM treatment or physical restraint, or covertly crushed CLO administered in food or drink without the patient's knowledge.

We considered articles meeting the following inclusion criteria: (i) published in English or French in peer‐reviewed journals; (ii) studies including people prescribed involuntary CLO treatment; (iii) reviews, editorials and letters addressing issues related to involuntary CLO treatment. These broad inclusion criteria were chosen in order to identify the clinical characteristics and the outcomes associated with prescribed involuntary CLO treatment, and to capture the legal issues and ethical debates raised by involuntary CLO treatment.

We did not consider articles: (i) exploring characteristics associated with CLO refusal in people without impaired capacity to make health decisions and hence not leading to involuntary CLO treatment [11]; (ii) focused on users' capacity to make healthcare decisions in general and not specifically to consent to CLO treatment [39, 40].

### 3.2 Study Selection

Titles and abstracts of retrieved citations were screened, selected full‐text articles were assessed for eligibility, and data were extracted independently by two researchers. Disagreement was resolved by discussion. We extracted the following methodological information: (i) author's name, publication year, country; (ii) study design and setting; (iii) demographic characteristics and diagnoses of CLO users; (iv) clinical context leading to involuntary CLO treatment; (v) modalities of involuntary CLO treatment: administration route (oral, IM, nasogastric) and procedure; (vi) main findings regarding the clinical outcome after the prescription of involuntary treatment, including statistically significant associations when reported. The data were synthesized narratively, since the literature mostly includes case reports or chart reviews, with little quantitative information.

## 4 Results

### 4.1 Literature Search

**FIGURE 1:**
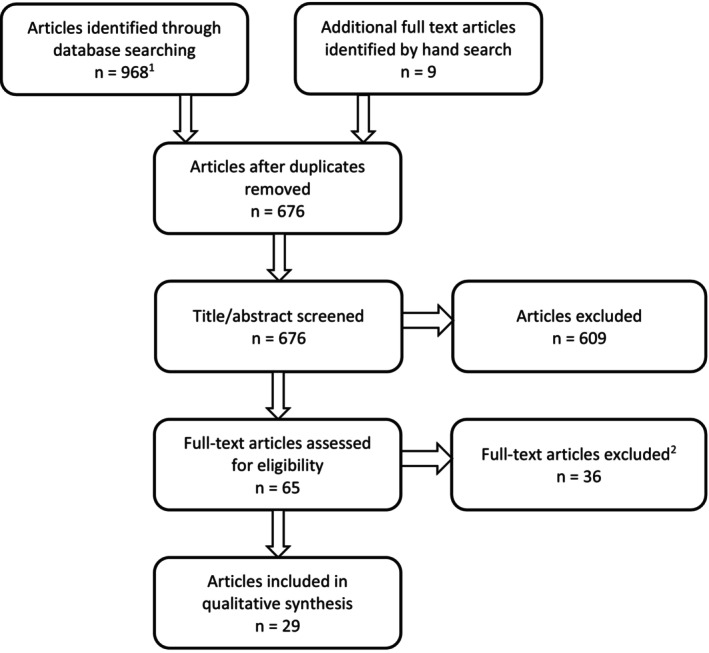
Study selection. (1) MEDLINE *n* = 335; Web of Sciences *n* = 376, PsycINFO *n* = 257. (2) No data on involuntary clozapine administration (*n* = 33), other unrelated topics (*n* = 3).

The flow chart of the eligibility process for this review is presented in Figure 1. We included 29 articles published from 1989 to 2024. The 18 studies on people prescribed involuntary CLO treatment (*n* = 236) are described in Table 1: nine case reports (*n* = 11 cases) [14, 16, 19, 33, 36, 41, 42, 43], three case series (*n* = 23) [17, 28, 32], four retrospective chart studies (*n* = 115) [15, 18, 44, 45], one population‐based study (*n* = 87) [27] and one intervention study [46]. They were conducted in nine countries: 14 (74%) were European (UK *n* = 9, Denmark *n* = 1, Germany *n* = 1, Netherlands *n* = 1, Serbia *n* = 1), the others were from USA = 2, Australia *n* = 1, India *n* = 1 and Israel *n* = 1.

**TABLE 1:** Studies on prescription and administration of involuntary clozapine (CLO) treatment.

Authors (year)	Type of study Setting	Age (years) Gender Diagnosis	Clinical context	Modalities of involuntary CLO treatment	Outcome and other main findings
Case report, case series and retrospective chart review studies					
Grohman et al. (1989) Germany	Case report (*n* = 1) from a chart study University psychiatric department	31 F^a^ SZ^b^	Psychotic exacerbation after 2 years of CLO Surgery for interatrial septal defect 13 years ago	IM administration	Cardiorespiratory arrest 4 h after IM^c^ CLO 100 mg and oral haloperidol 10 mg, died 4 days later
Durand et al. (1992) USA	Case report (*n* = 1) Massachusetts Mental Health Center	31 F SZ	Assaultiveness, 1 year in seclusion, several months in restraint	Forced drug delivery (nasogastric?) Court authorization, several staff meetings	− After decision of forced administration, “patient usually willing to take CLO” − Dramatic improvement over 9 months, became friendly and sociable, left the hospital for visits with family members
Marinkovic et al. (1994) Serbia	Retrospective chart study (*n* = 100 CLO users, no data on cases with IM CLO) (period NS) Institute of psychiatry, Belgrade	NS^d^ NS SZ	NS	IM administration	Tachycardia “commonly observed” immediately after IM ≥ 50 mg
Lokshin et al. (1999) Israel	Retrospective chart study (*n* = 59) All cases treated with IM CLO, Beer Sheva Mental Health Center (1993–1997)	Mean 37.6 M^e^ 67.8% NS	CLO initiated for at least a few weeks, non‐compliance to oral CLO, exacerbation of psychotic symptoms	IM administration	− Rapid sedation, improvement in behavior and cooperativeness permitting return to oral treatment within 3 days 27%; 4–7 days 71%; 8 days *n* = 1 − Infiltration at injection site *n* = 3 − 90% became compliant to oral CLO
Pereira et al. (1999b) UK	Case reports (*n* = 2) illustrating a “locally devised structured decision process for enforcing CLO therapy” Oxleas NHS Trust	37 M SZ	CLO refusal after 5‐year stabilisation, assaultiveness requiring seclusion and restraint	Oral administration IM neuroleptic if CLO refusal and behavior at risk for others Treatment plan discussed with multidisciplinary team, SOAD^f^ approval, family and trust managers informed	NS
		36 F SZ	CLO refusal, abnormal cervical smear test, refused somatic exams, intensive unit care for 1 year		NS
Mclean and Juckes (2001) Australia	Case report (*n* = 1) NS	64 F SZA	Non‐compliance to oral CLO, non‐responsive to ECT^g^	IM administration	− Oral CLO after 30 days − painful injections
Pfizer and Andrade (2002) India	Case report (*n* = 1) Outpatient	30 M SZ	CLO refusal 1 month after initiation ECT refused by family	Covert oral administration	Improvement maintained after 18 months of covert treatment
Schulte et al. (2007) Netherlands	Retrospective chart study (*n* = 17) Cases with enforced CLO identified by the members of the Dutch Clozapine Collaboration Group (1998–2003)	Mean 40.6 M 59% DSM^h^ IV SZ and SZA^i^	All in close wards, 7 in seclusion rooms; serious suffering, suicidality, self‐mutilation, assaultiveness, self‐neglect, anorexia, risk of permanent hospitalization	IM administration Enforced administration decision after discussion with the patient, his/her family and the nursing staff	− No IM needed: 41% − If IM needed duration < 4 days *n* = 4; 7–11 days *n* = 3; 1–3 months *n* = 3; physical restraint: 18% − Adverse effects: local swelling *n* = 2 − Follow‐up (mean 15.7 months): CLO maintained: 64.7%; CGI‐S^j^ decrease from 6.4 to 4.5 at (*p* < 0.0001); reduction of custodial restrictions: 65% − Motives for stopping IM: continuous IM CLO over 90 days, no perspective of a switch to oral CLO (*n* = 1); leucopenia (*n* = 1); impaired liver function (*n* = 1); psychic/somatic deterioration, local swelling (*n* = 1); recovery, convinced the commission for complaints to stop enforced treatment (*n* = 1); stop hunger strike and choose compulsory treatment with haloperidol decanoate (*n* = 1)
Silva et al. (2017) UK	Case report (*n* = 1) Ashworth high secure hospital	40s M SZ	Psychotically driven assaults, several years in seclusion, disheveled, long periods neither eating nor drinking, major weight loss involuntary ECT: as restrictive and invasive, greater health risk	Nasogastric administration SOAD approval, extensive discussions during multidisciplinary team meetings and with other professionals, consultation of family, social worker, patient's lawyer and independent mental health advocate	− Enforced CLO initiated for a 5‐day trial, extended in light of rapid improvement, 4 episodes over 19 days − Restrained by staff during insertion of nasogastric tube and CLO administration (15 mn) − After day 20, oral CLO, end of seclusion, marked improvement, transferred to a low‐security hospital after 7 months, then to rehabilitation hospital
Holmes (2019) UK	Case series (*n* = 7) All cases with prescribed IM CLO in Nottinghamshire Healthcare NHS^k^ Foundation Trust (period NS)	25–46 4 M, 3 F SZ, SZA, BP^l^, personality disorder	Previous CLO use *n* = 5 IM CLO for treatment initiation or because of intermittent adherence	IM administration	− No IM needed *n* = 4 − If needed: IM number *n* = 1–4, restraint *n* = 2 − All transferred or maintained on CLO and “have improved at least somewhat”
Till et al. (2019) UK	Case series (*n* = 5) All cases with nasogastric CLO administration, Ashworth high‐security hospital (2010–2016)	33–45 NS ICD‐10^m^ SZ	CLO already started *n* = 3 Confined to their rooms because risky to others, considered to be among the most extremely ill patients ECT unresponsive *n* = 1	Nasogastric administration SOAD approval, consultation of families, advocates and/or legal team	− No nasogastric tube needed *n* = 2 − If needed 1–4 episodes − 12‐Month follow‐up: clinical improvement, reduced number of incidents, termination of segregation (*n* = 4) − 24‐Month follow‐up: all accepted oral CLO
Casetta et al. (2020) UK	Retrospective chart study (*n* = 39) All cases with prescribed IM CLO, detained under the Mental Health Act in South London and Maudsley NHS Foundation Trust (2016–2019) Control group (*n* = 162): historical cohort of people with oral CLO initiation detained under the Mental Health Act	Mean 46 M 56% ICD‐10 SZ *n* = 18, BP/SZA *n* = 21	Previous CLO use: 82% Oral CLO refusal; short‐term intervention to initiate or re‐initiate CLO	IM administration choice offered with oral administration Multidisciplinary decision, SOAD approval	− No IM needed: 51% − If IM median number = 2, range 1–56, during titration period: 84%; manual restraint: 47% − Adverse effects: pain/swelling at injection site − Transition to oral CLO: no IM needed: 92%; at least one IM: 84% − Discontinuation rates at 2‐year follow‐up: no difference IM (24%) vs. oral CLO (50%) control group (aHR^n^ 0.39, 95% CI 0.14–1.06); higher if at least one IM (62%) vs. none (6%) among those with IM prescription (aHR 10.34, 95% CI 1.26–84.70)
Henry et al. (2020) UK	Case series (*n* = 11) All cases detained under the Mental Health Act in two medium‐secure units (*n* = 2), high‐secure hospitals (*n* = 2), locked rehabilitation unit (*n* = 1) (2017–2018)	Mean 37 M 100% ICD‐10 SZ *n* = 10, BP *n* = 1	“most” CLO responders, discontinuation for patient or medical reasons	IM administration choice offered with oral administration	− No IM needed *n* = 3 − If IM number of doses 1–99, restraint *n* = 7, no IM after 14 days *n* = 6 − Most often gluteal, max 250 mg in 10 mL, 3 injection sites − Adverse effects: none serious − 5‐Months follow‐up: oral CLO *n* = 9
Whiskey et al. (2020) UK	Case report (*n* = 1) Locked rehabilitation unit	51 M SZA	CLO responder, discontinued for heart failure, severely psychotic, suicide attempts, self‐neglect, violence, refused cardiology investigations	Oral/IM administration titration regime Multidisciplinary decision of CLO rechallenge	Considerable improvement in mental state, accepted cardiological exams
Gee et al. (2021) UK	Case reports (*n* = 2) Liaison psychiatry	50 F SZA	Stable on CLO for years, emergency admission for abdominal pain, refusal of CLO reintroduction and liver biopsy to rule out metastases, worsening of psychotic state and deterioration of physical health	IM administration	− IM CLO titration over 1 week − Deltoid injection because of morbid obesity − Marked improvement of psychotic symptoms within a few days, accepted oral CLO and medical investigations
		47 M SZ	CLO stopped for several months, oral CLO refusal, assaultiveness, suicidal attempt by jumping with severe injuries, high risk of aspiration because of spinal fracture	Nasogastric and IM administration “Swift communication between medical and psychiatric teams”	− CLO by nasogastric tube, removed the tube and refused remplacement − IM CLO leading to rapid improvement in mental state and deglutition allowing switch to oral CLO
Thapaliya et al. (2024) UK	Case report (*n* = 1) Acute psychiatric ward	40 F SZA	Stable on CLO for 11 years, discontinuation for thrombocytopenia, catatonia not improved with ECT, hospitalized > 9 months, refusal of oral CLO reintroduction	IM administration Prescription of oral CLO with option of IM CLO if refusal multidisciplinary decision, with 2nd opinion on CLO reintroduction from the Complex Psychosis Services Unit	− 1st 2 weeks of titration: *n* = 10 IM, last dose day 15 − Clinical improvement − Adverse effects: pain at injection site
Population‐based study					
Andersen et al. (2013) Denmark	3078 SZ patients with involuntary treatment identified using the Danish Psychiatric Central Research Register & the Registry of Coercive Measures in Psychiatric Treatment (2004–2010)	CLO: NS All group: mean 34.4 57% M ICD‐10 SZ	Involuntary treatment of psychosis or a life‐threatening medical condition (Coercive Protocol 2) Rapid tranquilization for severe agitation in emergency settings (Coercive Protocol 3)	Oral, IM, and nasogastric administration	− Oral CLO *n* = 85 (4.8% of all antipsychotics) − IM CLO *n* = 2 − Nasogastric tubes “mainly used for CLO” < 1%
Intervention study					
Fisher (2003) USA	Restraint and seclusion reduction program State Hospital, Creedmoor Psychiatric Center (1999–2001)	NS NS NS	CLO “aggressive use” is a key element of the program's pharmacological interventions if CLO refusal and persistence of highly dangerous behavior	Nasogastric administration Court orders to administer CLO over objection, even administering the first doses by nasogastric tube if necessary	− “Almost all” cases willing to continue CLO use “once the effects became evident” − Global program impact: 67% decline in seclusion/restraint rates

^a^ F: female.

^b^ SZ: schizophrenia.

^c^ IM: intramuscular.

^d^ NS: non‐specified.

^e^ M: male.

^f^ SOAD: second opinion approved doctor.

^g^ Electroconvulsive therapy.

^h^ DSM: Diagnostic and Statistical Manual of Mental Disorders.

^i^ SZA: schizo‐affective disorder.

^j^ CGI‐S: Clinical Global Impression‐Severity Scale.

^k^ NHS: National Health Service.

^l^ BP: bipolar disorder.

^m^ ICD: international classification of diseases.

^n^ aHR: adjusted hazard ratio.

The other articles (*n* = 11) were one systematic review [47], three reviews [34, 35, 48], one survey on practitioners' prescribing practices [49], one editorial [50] and five letters/reply to letters [37, 51, 52, 53, 54].

### 4.2 Characteristics of People Prescribed Involuntary CLO Treatment

Of the cases with available data on gender (*n* = 145), 92 (63.4%) were male. The mean age was around 40 years (range 25–64). Most studies included only people with schizophrenia spectrum disorders. Two also included people with bipolar or personality disorder [18, 32]. Information on diagnoses was missing in two studies [45, 46].

### 4.3 Treatment Resistance and Prior History of CLO Treatment

When information on history of prior treatments was detailed, it always indicated that prior pharmacological strategies failed, although the definition of resistance was not operationalized in all studies. Resistance to electroconvulsive therapy (ECT) was specified in two studies [17, 36] and another mentioned ECT refusal by the family [43]. In the case reported by Silva et al. [16], involuntary ECT was considered as more restrictive and invasive as involuntary CLO treatment and associated with greater health risk.

Prior positive response to CLO was documented in a minority of cases (*n* = 11) who discontinued CLO for serious medical reasons [19, 33, 36] or for other reasons [28, 41, 49]. At least three patients were in the titration period [17, 43]. At least 14 patients were CLO‐naïve [16, 17, 18, 32, 55]. The information on CLO treatment history was not detailed in the other studies.

### 4.4 Clinical Context Leading to Prescription of Involuntary CLO Treatment

All people were admitted to psychiatric wards, except two liaison psychiatry patients [19] and one outpatient [43]. Involuntary CLO treatment decision was motivated by refusal to initiate, reinitiate, or continue oral CLO. Reasons for refusal, which were not always explicitly documented in the studies [51], were often related to an impaired capacity to make an informed decision about optimal treatment because of refractory psychotic symptoms (poor insight, delusional beliefs about the treatment, etc.).

The most frequent target symptoms were risky behaviors: chronic assaultiveness [14, 15, 16, 19, 33, 55], suicidality, self‐mutilation, self‐neglect, or anorexia [15, 16, 19, 33]. A history of prolonged or iterative episodes of seclusion or restraint was often reported [14, 15, 17, 55]. Two patients refused physical exams required for potentially life‐threatening conditions [19, 33].

Involuntary CLO treatment was most often identified as the last‐resort treatment, as illustrated by the title of the article “when all else fails” [14]. The extremely severe and worrying clinical conditions leading to the decision to administer involuntary treatment were consistently reported in the studies. Patients were described by Till et al. [17] as *“to be among the most extremely ill patients*” and by Schulte et al. [15] as “seriously deteriorating under circumstances considered to be inhuman.” This worrying condition is summarized in the case reported by Silva et al. [16]: “*everyone was concerned about Ben's desperate condition*.”

### 4.5 Routes of Involuntary CLO Administration

The most frequent modality was the IM route, which was reported in 10 (55.6%) studies [15, 18, 28, 32, 33, 36, 41, 42, 44, 45]. Nasogastric CLO administration was reported by three studies [16, 17, 46] and oral administration by two [14, 43]. Two studies reported several routes of administration [19, 27] and the route was unclear (nasogastric?) in one study [55].

#### 4.5.1 IM CLO Administration

After CLO reintroduction in the late 1980s, the IM formulation (50 mg/2 mL) was produced by Novartis and initially licensed in several European countries and Israel, but the manufacturer discontinued it in 2005 for economic reasons [34, 35]. This formulation is currently marketed by Apotheek A15 in the Netherlands and by El Saad Pharma in Syria [34, 35]. In Europe, injectable CLO manufactured in the Netherlands is an unlicensed product imported to a few countries (e.g., Norway, Spain, Sweden, UK) after approval by the health authorities [28].

In the IM formulation, the CLO concentration is 25 mg/mL, i.e., a 5 mL ampule contains 125 mg [16, 34, 35]. The IM dose should be half the dose of oral CLO to take the 30%–50% lower bioavailability of the oral formulation into account [18, 28, 34, 35]. Although there is no published data on the pharmacokinetic of IM CLO, transiently higher CLO plasma levels can be expected with IM compared to oral administration [34]. The initial dose recommended by the South London and Maudsley (SlaM) protocol is 0.25 mL (i.e., 6.25 mg) [50].

Vigilance is required to ensure that the right dose is administered, since injection of the full 5 mL vial (equivalent to 250 mg oral CLO) can be lethal in CLO‐naïve people [28, 32, 50]. The injection should be administered in the gluteal muscle, but deltoid injection in a patient with morbid obesity has been reported [19, 35]. One daily dose is preferred [18]. As for other IM antipsychotics, there is a theoretical risk of accidental intravenous administration [16].

Since the administration of a large volume can be painful, at least two injection sites should be used if the dose is > 100 mg [18, 35]. The use of three injection sites has also been reported [28]. The SLaM protocol suggests not exceeding 14 days of injections [18] but administration over periods up to 3 months has exceptionally been performed [15, 18, 28, 42].

In spite of its higher bioavailability, IM CLO treatment is limited by the maximum deliverable dose (250 mg in 10 mL), and a higher‐strength solution for injection would facilitate titration and maintenance treatment when high doses are required [16, 18, 28, 35].

#### 4.5.2 Nasogastric CLO Administration

Involuntary nasogastric administration of psychotropic medication in psychiatric settings is rare [35]. In the Danish population‐based study carried out on schizophrenia inpatients receiving involuntary treatment, its frequency was < 1% and was “mainly for CLO” [27]. It requires restraint and is considered more burdensome for the patient than IM administration [16, 17, 28]. Different brands of CLO can be used [17]: licensed solutions (not available in all countries), unlicensed suspensions, or crushed tablets mixed in water. There is no published data on the pharmacokinetics of nasogastric CLO administration, but it is expected to be comparable to that of oral administration, so similar doses may be administered [17, 35]. The medical and nursing staff should be trained in safe nasogastric tube placement [17]. No radiological confirmation is required and the tube can be immediately removed after administration. The procedure described by Silva et al. [16] lasted up to 15 min. In a case of nasogastric administration in a patient intubated in an intensive care unit after a suicide attempt, the patient removed the tube and refused its replacement. CLO was then administered IM since the patient's difficulty in swallowing would have made oral administration unsafe [19].

#### 4.5.3 Involuntary Oral CLO Administration

In the Danish population‐based study, the most frequent involuntary route was oral with 85 cases (4.8% of all antipsychotics) vs. two for IM [27]. According to the procedure described by Pereira et al. [14], the patient should be aware that the drink contains CLO suspension in liquid form.

A survey on strategies for dealing with refusal to take oral CLO or blood tests that was addressed to UK consultants in intensive care units (*n* = 135, participation rate 31%) found that involuntary CLO administration was discussed in less than one out of three units [49]. The most common strategy for oral administration was to “offer inducement,” e.g., social activities (*n* = 5 cases), money or cigarettes (*n* = 2), stopping IM (*n* = 1). Negative reinforcement was also used with an IM antipsychotic whenever oral CLO was refused (*n* = 6). Other strategies included crushed CLO administered in drink with (*n* = 5) or without (*n* = 1) the patient's knowledge. Another case of covertly crushed CLO administered in food was peculiar, since CLO was obtained without prescription by the patient's family and was administered over at least 18 months without blood monitoring [43].

### 4.6 Legal Issues

#### 4.6.1 Involuntary Drug Administration

Regardless of the administration route, involuntary CLO treatment is regulated by each country‐specific legislation, thereby allowing involuntary drug administration for people with impaired capacity to make a decision about optimal treatment. For instance, people detained under the Mental Health Act in the UK may be administered coercive medication for longer than 3 months if a second opinion appointed doctor (SOAD) approves the treatment and the administration route [16, 18, 28]. The Danish Mental Health Act distinguishes two protocols [27]: Coercive Protocol 2 concerns involuntary treatment for psychosis or a life‐threatening medical condition, allowing recurrent administration until the condition has been cured or the patient accepts the treatment; Coercive Protocol 3 concerns rapid tranquilization for severe agitation in emergency settings.

In the USA, the reintroduction of CLO in the 90s impacted appellate‐level decisions on forced antipsychotic treatment [48]. Approvals of involuntary CLO administration were motivated by its higher efficacy compared to other antipsychotics. The appeals court also approved nasogastric administration: “*If the medication is medically necessary, the means to administer it must be medically necessary as wel*l” [48].

#### 4.6.2 Administration of Unlicensed Product

This issue mainly concerns IM CLO administration, since licensed CLO solutions or crushed CLO tablets are usually used for the nasogastric route [16, 35]. Country‐specific procedures also apply for the administration of unlicensed products. In the UK, the decision to prescribe unlicensed medication may be considered for last‐resort effective treatment whose benefits outweigh the risks [36]. Local protocols have been developed to allow unlicensed IM CLO administration [18, 19, 28, 34, 35, 50]. For instance, in London, the SLaM Drugs and Therapeutics Committee approved IM CLO prescription on an individual basis in 2016, where the agreement of the multidisciplinary team, the Director of Pharmacy and a SOAD were required [18, 28]. Regardless of country‐specific legislation, it is highly recommended to fully document the rationale for involuntary CLO administration in clinical notes and a care plan [28].

### 4.7 Decisional Process and Ethical Issues

The decision to prescribe involuntary CLO treatment usually generates much emotion and debate among staff members, who are frequently unfamiliar with involuntary CLO administration [17, 18, 19, 36, 51, 55]. Multidisciplinary staff meetings are often required before reaching a consensus, in order to weigh the risks and benefits of not acting vs. using coercive measures [16, 18, 19, 36, 55]. The decision should be discussed with the patient whenever possible, as well as with his/her family or legal representatives, carers, and advocates [15, 17, 18, 19]. The decisional process is often long, taking 6–8 months in 44% of cases in the study by Schulte et al. [15].

The ethical debates raised by involuntary CLO administration have been addressed in depth by authors with extensive experience of involuntary CLO treatment [14, 16, 17, 18, 19, 35, 37, 49, 54]. Some professionals may emphasize the right of all people to express their “will and preference” and therefore to refuse treatment, while other professionals may emphasize the right of people with severe psychotic symptoms to receive optimal treatment when they lack the capacity to make therapeutic decisions.

Involuntary CLO administration may be considered as punitive [14] and the need for restraint as “*degrading and inhumane*” [16]. In the case reported by Silva [16], some professionals suggested that it would be better to let the patient deteriorate to the point of life‐threatening dehydration in order to justify using a nasogastric tube to administer CLO. Debate has sometimes been heated [50, 51, 52, 53, 54]. For instance, a letter entitled “*Martial arts for psychiatrists*” [52] qualified the article by Pereira et al. [14] as “*instructions on how to restrain and overcome protesting patients and force them to take clozapine therapy*” and concluded “*I am concerned that this article was published at all, since it could be interpreted as incitement to violence*.”

Conversely, other professionals consider it unethical not to transiently use coercive measures if this is the only way to administer a treatment with well‐established efficacy for psychotic symptoms resistant to other treatments [15, 16, 17, 18, 19, 35, 37, 49, 54]. This position is easier to justify when the expected benefits are likely to be clinically significant, as for people with life‐threatening conditions or resistant assaultiveness that leads to enduring restraint or seclusion [16, 17, 18, 19, 35, 49, 54]. The impact of assaultive behavior on other patients and staff members should also be taken into account [14].

There is greater consensus regarding the fact that covertly crushed medication administered in food or drink without the patient's knowledge is considered unethical [43, 49].

### 4.8 Outcome After Prescription of Involuntary CLO Administration

#### 4.8.1 Actual Involuntary IM or Nasogastric Administration

The usual procedure is to prescribe involuntary administration while offering each time the “choice” of oral administration [17, 18, 28, 36, 37]. Actual IM or nasogastric CLO administration is much less frequent than might be expected [15, 17, 28, 35, 46, 55]. For instance, Schulte et al. [15] reported that no IM was needed in 41% of cases after the decision to perform involuntary administration. A comparable proportion was reported in the other studies [17, 18, 32]. In this context, oral CLO administration is also involuntary, at least initially, since the patient is informed that the treatment will be administered irrespective of his/her refusal.

The high rate of oral administration in people who previously refused this route often over weeks or months may be explained by the staff's conviction that CLO treatment is the only remaining option [15, 16], although the patient's fear of restraint and involuntary IM or nasogastric administration is likely to play a key role [17].

#### 4.8.2 Duration and Number of Involuntary CLO Administrations

For people who actually needed IM or nasogastric CLO administration, the rapid transition to oral administration was common [15, 16, 18, 28, 32, 34, 35, 36, 45, 46]. In most cases, it occurred within 7–14 days after 1–4 involuntary administrations [15, 16, 17, 18, 32, 45]. The highest duration was reported in a case with continuous IM CLO over 90 days before stopping, since there was no prospect of switching to oral CLO [15].

The proportion of injections or blood monitoring requiring physical restraint is not detailed in all studies. However, when reported, it was most often necessary in less than half of the patients (18% [15], 28% [32], 47% [18], and 64% [28]). For nasogastric administration, manual restraint by the staff was sufficient [16, 17].

#### 4.8.3 Adverse Effects

The most commonly observed adverse drug reactions (ADR) after IM administration are pain and swelling at the injection site [15, 35, 36, 42, 45]. Local abscesses have been reported with large injection volumes [45], justifying the use of at least two injection sites for doses > 100 mg (4 mL) [18, 35]. No ADR specifically related to nasogastric administration has been reported. The risks are low if the nasogastric tube is inserted safely and the restraint is as short as possible [16, 17, 28].

No study has directly compared the incidence of ADR according to the CLO administration route [35]. The frequency of tachycardia, hypotension, and sedation may be higher with IM vs. oral administration due to transiently elevated blood levels [34, 35, 44], especially with doses ≥ 50 mg [44]. A fatal cardiorespiratory arrest occurring in the hours following the administration of IM CLO 100 mg and oral haloperidol 10 mg was reported in a patient with a history of a septal defect [41]. The frequency of other ADR, including severe neutropenia, appears similar to that observed with oral administration [35].

#### 4.8.4 Clinical Outcome

After CLO initiation, the severity of the target symptoms decreased dramatically in all clinical studies [15, 16, 17, 19, 32, 33, 35, 36, 45, 55]. The most striking impact was observed for the reduction in assaultiveness and subsequent coercive measures (restraint or seclusion room sometimes lasting months and even years) [15, 16, 17, 46, 55]. In other cases, the decision to prescribe involuntary CLO administration allowed medical investigations for potentially life‐threatening conditions [19, 33].

The study by Schulte et al. [15] is the only one to have quantified the impact of involuntary CLO treatment on symptom severity, showing a Clinical Global Impression‐Severity Scale decrease from 6.4 to 4.5 over a mean of 15.7 months. Only 18% of the patients were retrospectively negative about being prescribed involuntary CLO treatment.

The descriptions of the clinical outcome of people having received involuntary CLO administration are often moving, reflecting the emotional commitment of the team. For instance, in one of the cases reported by Gee et al. [19], while the medical investigations allowed by CLO initiation did not change the prognosis, the authors concluded: “*although her cancer was ultimately sadly untreatable, her final weeks were spent in a calm, dignified and unrestricted way with her family*.” Another example is the description by Silva et al. [16] of a patient who spent several years in seclusion in a high‐security hospital: “*when he left, he thanked the care team for looking after him, giving both them and his treating consultant a thank you card. He remained in telephone communication with the ward nursing staff for over a year*.”

#### 4.8.5 Continuation of CLO Treatment

Studies including follow‐up after the acute phase found that CLO treatment was maintained in the majority of people who had received involuntary CLO treatment: 67% at the last observation (mean 15.7 months) [15]; 76% at 24 months [18]; 82% at 5 months [28]; 100% at 24 months [17]. In the study by Casseta et al. [18], discontinuation rates did not significantly differ between the group with involuntary CLO prescription (24%) and a historical control group of people with oral CLO initiation (50%) also detained under the Mental Health Act (adjusted Hazard Ratio, aHR 0.39, 95% CI 0.14–1.06). However, among those with an IM prescription, discontinuation rates were higher for people who needed at least one IM administration (62%) versus none (6%) (aHR 10.34, 95% CI 1.26–84.70).

## 5 Discussion

### 5.1 Main Findings

We identified 29 articles on involuntary CLO treatment published since 1989. Most clinical studies (*n* = 18 including 236 patients) were case reports, case series, or retrospective chart review studies. Most often, involuntary CLO administration was identified as the last‐resort treatment for people with extremely severe psychotic disorders presenting with risky behavior. The decisional process was often lengthy and complex since legal and ethical issues had to be addressed before reaching a multidisciplinary staff consensus on the best‐interest decision. In nearly half of cases, prescription of involuntary IM or nasogastric administration was sufficient to persuade the patient to accept the oral route. When IM or nasogastric administration was needed, transition to the oral route was often prompt. Pain at the injection site was the most frequent ADR observed after IM administration, justifying the use of several injection sites for high doses. A dramatic reduction in the severity of target symptoms and subsequent coercive measures (restraint, seclusion room) is consistently reported in the clinical studies. Follow‐up studies found that CLO treatment was maintained after the acute phase in the majority of people.

### 5.2 Limitations

Information on the benefits and risks of involuntary CLO treatment mostly comes from case reports and retrospective studies of small sample size. This limitation is inherent to the clinical profile of people requiring involuntary CLO administration who are unlikely to be included in controlled studies. Studies on the pharmacokinetics of IM CLO are also required since the current recommendations for IM doses are empirically based on a limited number of cases. The fact that IM CLO is currently available only in a few countries also restricts the possibility of large international studies.

Another limitation is that people prescribed involuntary CLO administration reported in the literature may not be representative of all people with such a prescription, as they may present with the most severe conditions at baseline and/or with a favorable outcome. However, several case series and retrospective chart reviews have attempted to identify all cases within a country or a mental health setting, limiting the impact of such selection biases [15, 17, 18, 28, 32, 45].

No cases of IM or nasogastric CLO administration have been reported in people with psychotic symptoms associated with Parkinson's disease or Lewy body dementia, both of which are also indications for CLO treatment [56]. It may be hypothesized that involuntary CLO administration in this population is instead carried out through undocumented covertly crushed medication mixed with food, a practice commonly observed in individuals with dementia [57, 58].

### 5.3 Implications for Clinical Practice

In clinical practice, coercive measures are exceptionally used to initiate or maintain CLO treatment. In the Danish population‐based study, involuntary CLO administration represented 4.8% of involuntary antipsychotics administration [27]. However, the frequency of involuntary CLO administration practices may be underestimated, especially for the oral route and in countries where no control exists for involuntary medication [31].

The scarce body of literature on involuntary CLO administration may be viewed as rather paradoxical. Indeed, the capacity to make health care decisions is frequently impaired by resistant psychotic symptoms. While antipsychotic IM administration is a current practice in psychiatric settings [23, 27], we need to understand why involuntary CLO treatment remains confidential and restricted to the most extremely ill people with SMI [14, 15, 16, 17, 18, 19]. People with resistant SMI associated with risky behavior are frequently exposed to recurrent IM administration of inefficient antipsychotics. Our objective is obviously not to promote the use of coercive measures, but to clarify why involuntary CLO administration should not be more controversial than treatment with IM olanzapine, haloperidol, or any other antipsychotics that are routinely administered involuntarily.

Several factors could account for the peculiar position of CLO among psychiatric treatments. The restricted access to the IM formulation, which is not available in most countries and is unlicensed in a few others, is a key limiting factor [28, 34, 35]. Another factor specific to involuntary CLO treatment is that further coercive measures may be required for blood monitoring [49]. Furthermore, the coercive context may exacerbate prescribers' barriers to CLO use [2, 3, 5, 59, 60, 61], leading to a greater overestimation of CLO risks and underestimation of its benefits. Lastly, initiating long‐term CLO treatment raises ethical questions different from those related to involuntary ECT, which is frequently short‐term [62]. Other factors may be less specific, such as questioning the impact of involuntary CLO treatment on future medication adherence and the therapeutic relationship [51]. Studies exploring this issue for all psychotropic drugs found that most people retrospectively considered that involuntary treatment was beneficial despite the negative feelings at the time of the coercive measure [15, 22, 63]. However, this interpretation is largely based on clinician‐reported outcome, since no study has systematically explored how patients experience involuntary CLO initiation, including perceived coercion, distress at the time of treatment, and retrospective reflections on benefits and harms. Qualitative and mixed‐methods studies would provide crucial insight into subjective experiences that cannot be inferred from clinical outcomes alone.

As a consequence of the limited access to IM CLO, it is currently restricted to a few extremely ill patients in a few countries. Hence, the target population of involuntary CLO treatment as a last‐resort option may be underestimated. Considering the dramatic impact of treatment‐resistant SMI, it may be relevant to address the question of how many people with resistant SMI are losing the opportunity to benefit from the only efficient treatment if involuntary CLO treatment is not prescribed. The target population may also be increased if licensed indications currently restricted to a few countries, such as suicidality, are extended to others.

Another key issue is how to assess the decision‐making capacity of people with a potential indication for involuntary CLO treatment. In cases reported in the literature, the assessment was based on the prescriber's clinical judgment, sometimes with a second opinion from another practitioner [16, 18, 28]. Structured assessment methods may prove useful to evaluate the different dimensions of decisional abilities: understanding information relevant to the healthcare decision that needs to be made, remembering it long enough to consider it in relation to one's own situation and to weigh up the benefit/risk balance, and being able to express one's choice [39, 40, 64]. For people already stabilized by CLO, psychiatric advance directives can allow users to express their will and preferences regarding CLO re‐initiation in the event of being unable to make a health decision after a period of CLO withdrawal [20, 25, 29, 65].

## 6 Conclusion

Despite the limitations of the studies on involuntary CLO treatment, their findings suggest that it may save the life and promote the recovery of people for whom all other treatments have failed. The restricted availability of the IM formulation may be considered a barrier to CLO treatment for some of the most severely ill people with SMI. This barrier is exacerbated by the relationship between supply and demand: if the majority of prescribers in a country are unaware that an IM formulation exists, there is no indication and hence no demand for it, so health authorities are unlikely to approve and initiate the procedure to obtain CLO ampules. Examples of European countries that have successfully overcome this barrier are extremely helpful [15, 16, 18] and could encourage the joint mobilization of other countries to facilitate access to the IM formulation.

## Author Contributions

**Hélène Verdoux:** conceptualization, methodology, investigation, writing – original draft, review and editing, project administration. **Alexis Lepetit:** methodology, validation, writing – review and editing. **Peter F. J. Schulte:** conceptualization, methodology, investigation, validation, writing – review and editing.

## Funding

Open access publication funding provided by Bordeaux University (COUPERIN CY26).

## Conflicts of Interest

The authors declare no conflicts of interest.

## Data Availability

Data sharing not applicable to this article as no datasets were generated or analysed during the current study.
